# Client Factors Affect Provider Adherence to Guidelines during First Antenatal Care in Public Health Facilities, Ethiopia: A Multi-Center Cross-Sectional Study

**DOI:** 10.4314/ejhs.v30i6.8

**Published:** 2020-11

**Authors:** Tewodros Seyoum, Mekuriaw Alemayehu, Kyllike Christensson, Helena Lindgren

**Affiliations:** 1 School of Midwifery, College of Medicine and Health Sciences, University of Gondar, Gondar, Ethiopia; 2 Department of Environmental and Occupational Health and Safety, Institute of Public Health, University of Gondar, Gondar, Ethiopia; 3 Department of Women's and Children's Health, Karolinska Institutet, Stockholm, Sweden

**Keywords:** Complete provider adherence, first ANC visit, Ethiopia

## Abstract

**Background:**

Timely entry to an antenatal care with a healthcare provider who follows a set of national guidelines is assumed to ensure higher levels of client satisfaction. It is also expected to improve perinatal outcomes. Little is known about the level of adherence of Ethiopian providers to these guidelines. Therefore, this study aims to assess the proportion of clients who received complete (100%) provider adherence to antenatal care guidelines at the first visit and client associated factors in Gondar Town.

**Methods:**

A cross-sectional study of 834 study participants was conducted in public health facilities of Gondar Town in Ethiopia. An 18 point checklist was used to observe provider adherence to the first antenatal care visit guidelines. Descriptive statistics and multivariable binary logistic regression model were done by using STATA 14 software.

**Result:**

The proportion of clients who received the complete provider's adherence to the first antenatal care guideline was 32.25% (95% CI: 29.1–35.5). The mean adherence score was 16.78%. Women who had prior history of pregnancy and/or birth-related complications (AOR = 1.58; 95%CI: 1.04–2.04) and late antenatal care booking at gestational week 16 or greater (AOR = 1.45; 95%CI: 1.03–2.03) were significantly associated with clients receiving complete providers' adherence to the first antenatal guideline.

**Conclusions:**

We found the level of adherence to national antenatal care guidelines during first visit as surprisingly low. When considering to upgrade the guidelines to the actual WHO guideline of eight visits, we recommend that refresher training be provided regularly to help staff understand the importance of following the guidelines as closely as possible. Perhaps, we need to learn more from the health care providers themselves about their reasons for not following the guidelines.

## Introduction

It is well established that there are steps that if taken can reduce maternal and perinatal mortality in low-income countries including Ethiopia. An essential first step is to increase the level of attendance by mothers to beat all four Antenatal Care Visits (ANC) that are part of the Ethiopian maternal care program. Maternal mortality has declined over the past few decades (1,2) and goals have been set stating that the global maternal mortality ratio should be less than 70 per 100, 000 live births by 2030. Maternal mortality continues to be far above that level worldwide. Meeting that goal remains as a major challenge (3).

According to the 2019 WHO estimates of the global maternal mortality ratio in 2017 there were 211 maternal deaths per 100, 000 live births. This represented a 38% reduction from the estimate reported in 2000, 342 per 100,000 live births. The sub-Saharan African region continued to have the highest maternal mortality in 2017, estimated at 542 deaths per 100, 000 live births, accounting for 66% of the total of global maternal deaths. Ethiopia was one of three countries in Africa that had 10, 000 maternal deaths or more (4).

A focused Antenatal Care (FANC) model has been used as a working guideline for more than two decades in Ethiopia (5–7). A review of the efficacy of FANC found that only four ANC visits increase perinatal deaths and decrease client satisfaction (8,9). Based on these and similar findings, WHO developed a new eight-contact ANC model in 2016, which aims to put women at the center of care (10). In Ethiopia, however, the four visit ANC model remains as the standard. A newly updated guideline has been formulated but has not as yet been implemented (11).

The Ethiopian Demographic Health Survey (EDHS) 2019 mini report showed that 74% of pregnant women attended at least one ANC visit but that only 48% of clients gave birth at a health facility (12). This disconnected coverage of services has fueled calls to focus on the quality of ANC services in Ethiopia. Process attribute is one of the gold standard dimensions to measure quality which assesses the content of the service delivered by the provider as per the ANC guidelines through direct observation (13).

WHO recommends that the first ANC visit be undertaken before gestational week 12. In Ethiopia, the national recommendation is that the first ANC visit should take place before gestational week 16 (6,10,11). The first ANC contact is a good point at which the healthcare provider can identify and distinguish pregnant women who are eligible to receive routine and standard care and to establish a program of continuum of care (11,14).

Timely and complete adherence by the provider to the protocol during the first ANC visit has been shown to increase the women's level of satisfaction, enhance their motivation to attend subsequent follow-up visits, increase the likelihood of institutional delivery, and improve perinatal outcomes (15–17). Nevertheless, the proportion of clients whose providers adhere completely to the guidelines at first ANC visit remains low (18,19). Several factors contribute to this low adherence to ANC guidelines. Limited awareness of current clinical guidelines is one; low availability of supplies and equipment is another, and high client loads a third (20,21). Client-related factors may also have contributed to a provider's low adherence to the guidelines, but this has not been extensively studied (19,22). There is also a paucity of evidence in Ethiopia on the extent to which providers adhere to the first ANC visit guideline. Therefore, this study aimed to assess the proportion of clients who received complete (100%) provider adherence to care guidelines at the first ANC visit and client associated factors in public health facilities in Gondar Town.

## Methods and Materials

**Study design, setting, period and population**: A cross-sectional study was conducted in Gondar Town public health facilities from May 12, 2019 to July 25, 2019. The total population size of the town is estimated to be 306,246. Of these, 156,276 were females in reproductive age (23). There are eight public health centers and one comprehensive specialized hospital in the town.

All women who came for routine ANC services at selected public health facilities in Gondar during the data collection period were considered for inclusion in the study

**Sample size and power**: The sample size was estimated by using single population proportion formula (24). The following assumptions were considered: proportion of clients who received complete adherence to care guideline of first ANC visit with the previous study done in Ghana was found to be 48.1% (19), level of significance to be 5% (α = 0.05), Z α/2 = 1.96, margin of error to be 5% (w = 0.05) and considering sufficient sample size for determinant factors. Considering design effect =2 and adding a 10% non-response rate, the final calculated sample size was 834.

**Sampling procedure**: Multistage sampling technique was employed to conduct this study. In the first stage, four health facilities (one hospital and three health centers) were selected among nine public health facilities by a simple random sampling technique. Then, a proportional allocation was done to each facility based on the average number of first-time ANC users in the most recent three-month reports. In the second stage, systematic random sampling technique with sampling interval of 2 was used to select the study participants. The first participant was selected by lottery method.

**Variables**: The outcome variable of the study was clients who received providers' adherence; the explanatory variables were type of facility, clients' socio-demographics, and client's obstetric factors.

## Measurement of Variables

**Providers' adherence**: Providers' adherence was defined as providers' “conformity to, fulfilling standard ANC guidelines as per the national protocols (25).

**Level of provider adherence**: Level of adherence was measured using a scoring system, based on available guidelines (14,19,26).

**Received complete provider adherence**: Clients who received all the 18 items adhered to by the provider as listed in care guidelines for the first ANC visit (total score = 18).

**Counseling**: Women who got at least one counseling service among the components of ANC during the first visit (Nutrition, PMTCT, birth preparedness and complication readiness, danger signs) were recorded as having received counseling (15).

**Data collection procedures**: Firstly, an 18 point checklist/observation protocol was developed based on the Ethiopian FANC guideline document (11,14). Second, a checklist was developed to assess/audit the facility's readiness for providing ANC service. In addition, a structured interview guide was prepared in English and then translated into Amharic and back into English to maintain consistency. Seven female midwife data collectors and three supervisors with master's degrees in clinical midwifery were, and training was given for three days by investigators. A pre-test was done outside the actual study area at Debark District hospital with 10% of the total sample size.

After pre-testing the three different tools, facility audit assessment was done and participants were enrolled at their first ANC booking once they provided informed consent for participating. Pregnant women who were to report for an ANC visit and had an age of 28 weeks or fewer were included as candidates to take part in the study.

After enrollment, a direct observation was done by data collectors to assess how the ANC services/contents were given as well as the information provided using the observation protocol. Face-to-face interviews were conducted to collect the clients' sociodemographic and obstetric data by using structured and standardized questionnaire. On-site supervision was conducted during data collection.

**Data processing and analysis**: All data were checked manually for completeness, coded and entered into Epi-info version 7 software and then transported to STATA 14 for analysis. Descriptive statistical scores were used to summarize the findings. Bivariate analysis was conducted to determine the crude association between dependent and independent variables. Those variables with P-value < 0.25 were entered into multivariable binary logistic regression. Statistical significance was declared at P-value <0.05.

**Ethical considerations**: The protocol was reviewed by the Institutional Ethical Review Board of the University of the Gondar for its ethical soundness, ID: O/V/P/RCS/05/498/2018. Signed informed written consent was received from each respondent before the data collection, and confidentiality was maintained.

## Results

**Participants' Socio-demographic characteristics**: A total of 4961 pregnant women were eligible for the study; 834 participants were recruited with a 100% response rate. Half (52.3%) of the participants were included from the hospital. The majority (82.49 %) of the participants were in the age group of 20–35 years with a mean (±SD) age of 25.92 ± 0.17 years. Almost all (97%) were married and urban residents (Table 1).

**Table 1:** Socio-demographic characteristics of first ANC attendants in Gondar Town public health facilities, 2019 (n = 834)

Socio-demographic variables		Frequency	Percentage
Type of health facility getting ANC service	Hospital	436	52.30
	Health centers	398	47.70
Maternal age (in years)	<20	11	13.91
	20–35	688	82.49
	>35	30	3.60
Place of residence	Urban	816	97.84
	Rural	18	2.16
Religion	Orthodox	765	91.73
	Muslim	59	7.07
	Others	10	1.20
Marital status	Married	810	97.20
	Unmarried	24	2.88
Maternal educational level	None	142	17.03
	Primary	206	24.7
	Secondary	256	30.7
	Tertiary	230	27.58
Maternal employment status	Employed	195	23.38
	Not employed	639	76.62

**Participants' obstetric characteristics**: Three hundred thirty (40%) of the participants were primigravidae and 377(45%) nullipara. One hundred eleven (14%) of the study participants had previous history of pregnancy and birth-related complications. Only 243(29%) of the participants came to their first ANC before or during gestational week 16 (Table 2).

**Table 2:** Obstetric profiles of first ANC attendants in Gondar Town public health facilities (n = 834)

Obstetric variables		Frequency	Percentage
Timing of first ANC booking	Early booking (<16 weeks)	243	29.14
	Late booking (≥ 16 weeks)	591	70.86
Gravidity	Primigravidea	330	39.57
	Multigravidea	504	60.43
Parity (number)	0 (nulliparious)	377	45.20
	1–2	379	45.44
	3–4	64	7.67
	>4	14	1.68
Previous history of pregnancy related complications	Yes	111	13.31
	No	723	86.90
length of birth to pregnancy interval (in month)	Short (≤24)	110	23.76
	Recommended (24–48)	178	38.44
	Long(>48)	175	37.00
Intention of the current pregnancy	Planned	762	91.37
	Unplanned	72	8.63

**Facility audit in the study settings**: At least five midwives were permanently assigned in each health facility. Medical interns and obstetricians were also part of maternal care units at the hospital and at one of the health centers. Access to the guidelines and training programs designed to teach providers to follow these guidelines were no complete programs in use.

All the studied health facilities had examination coaches, blood pressure apparatuses, and adult weight scales, reagents, hematocrit centrifuges, test kits for Venereal Disease Reference Laboratory (VDRL), HIV test and some essential drugs such as iron and folic acid tablets.

**The proportion of clients who received provider adherence to first ANC guidelines**: Six hundred and twenty (74%) of the participants were asked about their general medical history. During physical examination, 806(96.6%) participants had their blood pressure checked. A total of 817(98%) participants had hemoglobin test. About 783(93%) of participants had iron tablets prescribed; of these 569(72.6%) got the prescription after gestational week 16 (Table 3).

**Table 3:** Summary of clients who received provider adherence to first ANC guidelines, at Gondar Town public health facilities (n = 834)

Variable	category	Frequency	Percentage
1.	Age asked	766	91.85
2.	Gestational age calculated	832	99.76
3.	Parity asked	830	99.52
4.	Past obstetric history asked	799	95.80
5.	Previous abdominal-pelvic surgery asked or checked	707	84.77
6.	Medical general history asked	620	74.34
7.	Current pregnancy history asked	697	83.57
8.	Weight measured	806	96.64
9.	Blood pressure checked	819	98.26
10.	Abdominal examination has done	726	87.05
11.	Hemoglobin test has done	817	98.00
12.	Urine test has done	833	99.88
13.	VDRL has done	833	99.88
14.	Blood group and Rh have done	834	100.00
15.	HIV test has done	818	98.08
16.	Tetanus injection has given	817	97.96
17.	Iron folic tablets prescribed	783	93.88
18.	Counseling has done	663	79.50

**Figure 1:**
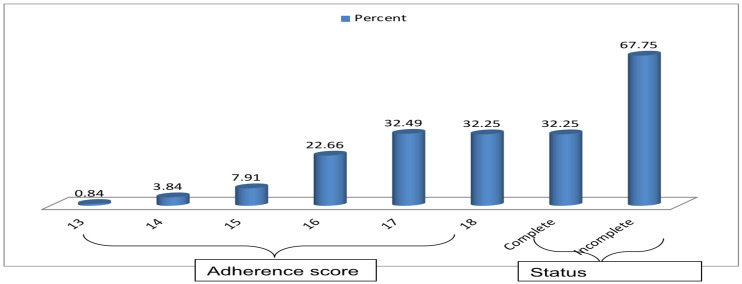
Score and level of clients received providers' adherence to first ANC guidelines for 834 study Participants, Gondar town public health facilities

The mean adherence score with 95% CI was 16.78(16.71–16.86) for all 18 items. About 23% of the participants scored 16 out of 18 items. The proportion of clients who received complete provider's adherence to the first ANC visit guidelines was 32.25% (95% CI: 29.1–35.5) (Figure 1].

**Client factors that affect to receive complete provider adherence to clinical guidelines**: In the bivariate logistic regression, history of pregnancy and birth-related complications, late ANC booking, primary and secondary educational level, being married and being women in the age group of 20–35 years were associated with clients who received complete providers' adherence to first ANC guidelines. In the multivariable binary logistic regression, only history of pregnancy and/or birth-related complications (AOR = 1.58; 95%CI: 1.04–2.04) and late ANC booking (AOR = 1.45; 95%CI: 1.03–2.03) were associated with clients who received complete providers' adherence to first ANC guideline (Table 4).

**Table 4:** The bivariable and multivariable binary logistic regression analysis of factors associated with clients received who complete provider adherence to first ANC visit guidelines (n=834)

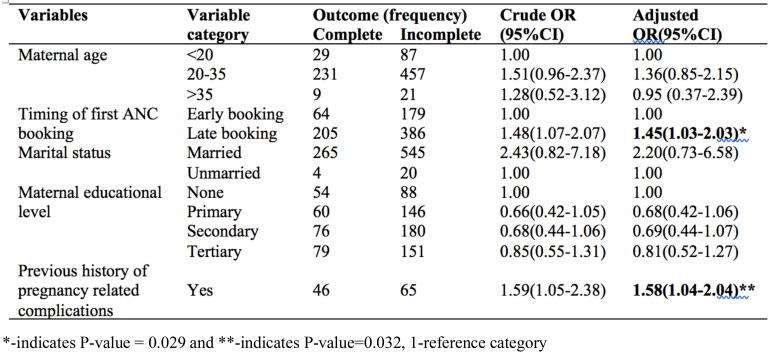

## Discussion

The main finding of this research revealed that only one-third of the clients received complete providers' adherence to the first ANC guidelines. At the same time, the mean adherence score was relatively high. Women with previous complications or late booking received complete adherence to a higher extent.

The proportion of clients who received complete providers' adherence is similar to the results from a study done in Bahirdar Town which reported that 27.6% received an acceptable quality of ANC services (15). The similarities and low overall adherence found in both studies might be explained by inadequate training of healthcare professionals in the use of the FANC guidelines. This possibility is supported by the results of the audit assessment done at all participating facilities, reporting that most providers did not receive in-service training on following FANC guidelines and that none of the health facilities had access to the ANC guidelines. The level of adherence found in this study is much lower than the level in studies done in Ghana (48.1%) and Tanzania (47.1%) (18,19). These differences could be due to differences in study design and measurement of the outcome variable. In contrast with the present study, the level of providers' adherence to the ANC guidelines in the Ghanaian study was determined retrospectively from maternal health record book. Therefore, it would be difficult to ascertain the outcome variable. The observed difference between the findings of our present study and the study conducted in Tanzania could be due to a difference in measurement of outcome variable. In Tanzania, clients receiving more than 60.5% cut score was considered as complete providers' adherence to the first ANC guideline. In contrast, the present study measured the level of clients to receive provider adherence in the first ANC guideline with all or none approach.

However, even if complete adherence was admittedly low (32%), mean adherence was 16.78 which must be seen as quite high. There were variables which contributed to the high mean adherence score like blood-group test, hemoglobin test, and blood-pressure measurement. Findings for these items were consistent with those of previous studies done in Ethiopia, Ghana, and Tanzania (15,18,19). The similarity between the results for these items very likely resulted because locations in all three countries had the same diagnostic tools and equipment. Providers in all three locations do make these measurements and carry out the tests. These are important measurements to be done in early pregnancy since they are needed to aid in detecting current and pre-existing conditions such as anemia and hypertension and thus can be taken into consideration in deciding on early treatment and counseling the woman and her relatives (7).

There were also items for which the adherence scores were very low. In contrast with results from the other studies (18,19), the results from the present study indicated that many Ethiopian providers did not report the medical history of the clients, thus the adherence score was lowest for medical history. The observed difference between the findings of our present study and the findings from other studies might be due in part to differences in ascertaining the variables to be measured. In the current study, the outcome variable was determined prospectively. Therefore, it was easy to ascertain the authenticity of the data. However, in the previous studies, the outcome variable was collected from client files retrospectively. Providers might write on clients' medical record books without asking them and this may contribute to high scores of some variables. Failing to detect pre-existing conditions such as hypertension, diabetic mellitus and urinary tract infection might result in a failure to take these conditions into account in creating a treatment plan. This could result in adverse outcomes for both the mother and the fetus (7). Another item for which the adherence score was low was “counseling of women regarding danger signs of obstetric complications”. Both the previous ANC guidelines and the new WHO ANC models recommend that counseling and assessment of danger signs should be started during the first ANC visit (10).

According to the present study, the odds of clients who booked their first ANC visit at gestational week 16 or later was 1.45 times more likely to receive complete provider adherence to ANC guidelines of the first visit. This finding is in contradiction with the WHO recommendation that states that every pregnant woman should initiate her first ANC visit before gestational week 12 and before gestational week 16 in Ethiopia's specific recommendation with appropriate service as per the guideline (6,10,11). The women who came at later gestational age had to be enrolled as making their first visit with the provider following the ANC first-visit guidelines but the provider was also allowed to choose to provide as services appropriate for a client at gestational week 16 or later (14). Women who register late may miss the opportunity to be given iron and folic acid tablets early enough to prevent anemia and congenital malformation (27). Previous studies have indicated that starting anemia treatment in the first trimester prevents bleeding during child-birth, low birth weight, and pre-term birth (7,28). However, 93% of the participants in the present study were prescribed iron tablets, but of these, almost two of three only began to take these at gestational week 16 or later. A similar finding was reported from Ghana (19). In both studies, healthcare providers may be reluctant to prescribe iron and folic acid tablets during the first trimester of pregnancy as they may worry about losing their clients due to the side effects of tablets (30).

Likewise, there is an observed positive correlation between late ANC booking and abdominal examination in the present study. Healthcare providers may perceive that an abdominal examination should be done first in the second trimester because the fetus is not abdominally palpable in the early gestational age (19). An abdominal examination can, however, assure the accuracy of the day of the last menstrual period and is useful in assessing fetal viability towards the end of the first trimester (31).

The providers who had clients with history of pregnancy and/or birth-related complications were more likely to follow the guidelines than were providers who had clients who came at the recommended or preferred time. Identifying clients who had previous obstetric complications early is important in order to provide specialized care in addition to the routine care. All clients should receive complete ANC service in the first visit (6,11). The finding that this is not true is troubling. If the 18 point/item checklist was designed for all first-visit clients, whatever their prior history, this checklist should be followed by all providers. The finding of the present study is similar to that described in a study done in Chicago (22). Healthcare providers give more emphasis to those clients who had previous pregnancy-related complications by providing guideline-specified ANC in the first ANC visit. In contrast with our finding, another study done in Ghana revealed that clients who had previous history of pregnancy and birth-related complications were less likely to have providers who adhered completely to care guidelines for the first ANC visit (19). In fact, in all studies, the findings are against the recommendation of focused ANC which aims to give holistic individualized, client-centered care and detect problems but not classify clients based on their level of risks (6).

In general, the finding of our present study will give an overall insight on the quality ANC for maternal healthcare programmers and implementers in Ethiopia and similar countries. Capacitating providers on how to adhere with the standards of ANC guideline in the first visit is very crucial.

The prospective design is the main strength of our study. Since this was a cross-sectional study with direct open observation, information bias was reduced and authenticity of data was maximized. The study has also a limitation. Healthcare providers might make maximum effort to carry out the first ANC visit if the data collectors were directly observing that visit (Hawthorne effect). However, the effect of an observer lasts only for a short time and it slowly decreases when the providers adapt to the presence of the observer (32). To minimize this bias, data collectors did not tell the ANC providers about what specific ANC services they were observing rather than telling them in a general way.

In conclusion, the proportion of clients who received complete providers' adherence to the first ANC guidelines was low although the mean adherence score was quite high. Late booking and having previous pregnancy and birth-related complications were the factors associated with having providers who adhered completely to the first ANC guidelines. We need to know much more from the providers themselves concerning their own reasons for not adhering to guidelines. It appears that the in-service training of providers needs to be improved and provided regularly but a program to make these improvements can only be based on a better understanding of the reasons for not following the guidelines. Such understanding cannot be learned from the clients; so, it appears that additional studies are needed that focus on the providers.
